# A Motivational Mechanism Framework for Teachers' Online Informal Learning and Innovation During the COVID-19 Pandemic

**DOI:** 10.3389/fpsyg.2021.601200

**Published:** 2021-03-31

**Authors:** Haiqin Yu, Jian Zhang, Ruomeng Zou

**Affiliations:** ^1^School of Education, Huazhong University of Science and Technology, Wuhan, China; ^2^School of Economics and Management, Beijing University of Science and Technology, Beijing, China

**Keywords:** motivational mechanism of innovation, informal learning, innovative teaching, personal teaching efficacy, autonomous motivation, online teaching and learning context

## Abstract

Online informal learning (IL) spreads quickly in the COVID-19 Pandemic. Studies have predicted that both online and workplace IL have potential value to individual and organization development, whereas the study on its link with innovation remains scarce. IL is an individualized learning pattern different from formal learning, and its functioning mechanism on innovation will deepen our understanding of the relationship between learning and innovation. Self-efficacy and autonomous motivation are considered as two streams of motivational mediating mechanisms to innovation. However, previous studies have proceeded largely in separation from each other. Researchers highlight the need to develop a more fine-grained theory of motivation and innovation. In addressing these literature gaps, this paper takes college teachers as the sample and focuses on the motivational mediating mechanism between online IL and innovation. The results showed that teachers IL could positively predict innovative teaching performance. Personal teaching efficacy and autonomous motivation played as sequential mediators on the link between IL and innovative teaching performance. This study extends the literature of IL–innovation relationship and enriches understanding of cognition-oriented motivation theory, highlighting one's internal autonomous construction is the key to innovation. Theoretical and practical implications for psychological empowerment are discussed.

## Introduction

Online education became ubiquitous due to the COVID-19 Pandemic during 2020 (Li et al., 2020; Quezada et al., 2020; Tian et al., 2020). However, making good use of the Internet for education remains problematic (OECD, 2020). Teachers need to learn and innovate in their work. For example, China's Ministry of Education required colleges to organize teachers to learn online teaching methods and provide some free and open teaching resources across regions and colleges (China's Ministry of Education., 2020) to cope with online teaching, which lasted half of the school year. As a result, some teacher communities of online informal learning (IL) formed throughout the country. Teachers' online IL usually includes selecting learning resources (video conference/seminar, reading), communicating with other teachers to solve problems, discussing ideas related to work, observing and analyzing teaching cases, exchanging information from different colleges, etc. IL represents a self-directed and social constructivist (Marsick et al., 2017) learning pattern in an informal context. Some studies have predicted that online IL and workplace IL have broad prospects in adult learning and organization management (Thomas, 2004; Yu and Mao, 2005; Jacobs and Park, 2009). However, research on its effects, particularly on innovation, remains scarce. In the fields of education and management, although the positive impact of learning on innovation has been extensively verified, the learning pattern is basically a well-designed formal learning project with structured content (Scott et al., 2004). These results show that innovation can be improved through learning and training. Thus, IL may be an improving force, too. However, IL differs in nature from formal learning. Its learning method is characterized by observation and imitation, cooperation and communication, and personal reflection, making it a highly spontaneous and personalized form of learning. We wonder whether IL has a natural connection with innovation that emphasizes individuality. This study's main purpose is to explore the possible effect of teachers' online IL on innovative teaching, which will enrich our understanding of innovation and expand the organizational approach to innovation intervention.

Over the past 30 years, studies of the underlying motivational mechanisms of innovation have generated valuable knowledge for researchers and practitioners. Self-efficacy and autonomous motivation (AM) are considered to be two important predictors of innovation, representing two mediating mechanisms that connect environmental factors to employee innovation (Liu D. et al., 2016), respectively, drawn from Bandura's (1997) social cognitive theory and Amabile's (1996) componential theory of innovation or Gagné and Deci's (2005) self-determination theory (SDT). Self-efficacy encourages the individual to engage in creative processes and maintain their level of involvement through belief in their ability to successfully accomplish these processes (i.e., “can-do” motivational force) (Tierney and Farmer, 2002). Meanwhile, AM propels the individual to devote their efforts to creative processes by arousing their interest in and enjoyment of work (i.e., “want-to” motivational force) (Amabile, 1993; De Jesus et al., 2013). However, these two streams of research on how motivation influences creativity have largely remained separate. Researchers have highlighted the need to develop a more fine-grained theory of motivation and creativity (George, 2007; Liu D. et al., 2016). In addressing this literature gap, this study's second purpose is to investigate the roles of personal teaching efficacy and AM in the association between online IL and innovative teaching. We expect to find that self-efficacy and AM are sequential mediators in this relationship. This research design is conducive to a complete explanation of the motivational mechanism through which IL affects innovation, expanding cognitive motivation theory.

### Informal Learning and Its Relationship With Innovation

Some researchers have insisted that most workplace learning occurs outside of formal training, with IL accounting for 70–90% (Flynn et al., 2006; Koopmans et al., 2006; Cunningham and Hillier, 2013). They recognized the potential value of IL for personal and organizational development. However, at present, there is no singular definition of IL or unified approach to its definition, largely due to the intersecting interests, contested ideas, and multiple approaches in the field (Manuti et al., 2015). The following four dimensions are well-rooted in the literature. (1) IL is always self-initiated and strongly intentional. It often occurs as people want to change themselves to meet current and future work requirements (Thomas, 2004; Jacobs and Park, 2009; Marsick et al., 2017). (2) IL is usually unplanned and loosely organized and occurs without any clear learning structure or outcome evaluation (Jacobs and Park, 2009; Marsick et al., 2017). (3) It is experiential learning, involving experimentation or new experience, e.g., seeking new assignments; doing a task differently (Wolfson et al., 2018). (4) It is learning through interaction and reflection, e.g., actively seeking feedback and advice and debriefing work experience (Manuti et al., 2015; Louws et al., 2017). In all, it is characterized by autonomous, random, problem-solving, and social constructivist learning. During the COVID-19 pandemic, it takes place at home and online. We conceptualize online IL as teachers' spontaneous engagement in learning activities organized by institutions or teachers themselves that permeate the daily lives of faculty.

Prior research on the impact of IL is limited and mainly focuses on employees' work outcomes, such as work performance, work attitude (job satisfaction, organizational commitment), and organizational adaptation (Rowden and Conine, 2005; Zhao and Gao, 2017; Wolfson et al., 2018), as well as teachers' competence (teaching knowledge, experience, attitudes, and strategies) (Kyndt et al., 2016; Louws et al., 2017). These studies explain the positive impact of IL from the perspective of interpersonal interaction. Other studies in the domain of innovation support this perspective, too, though only concerning some components of IL. For example, extensive feedback-seeking and alternative learning experience can directly give individuals innovation experience and domain skills, reduce costly mistakes and protracted search, broaden individuals' innovative ideas, and thereby improve individual and enterprise innovation performance (Gino et al., 2010; De Stobbeleir et al., 2011; Harrison and Rouse, 2015; Abecassis-Moedas et al., 2016). Similarly, it is found that practice communities and informal networks are concentrated bases of employee innovation for promoting their feedback-seeking, perceived organizational support, and knowledge management (transmission) (Wenger and Snyder, 2000; Eraut, 2004; Zhou and Lu, 2009; Liu X. Y. et al., 2016). The results mentioned earlier suggest there is a potential connection between IL and innovation.

Community-based online IL supported by internet technology is regarded as a typical form of IL (Yu and Mao, 2005). We think the differences between IL and online IL are the degree of individual autonomy, learning convenience and diversity, and, evidently, online IL has more advantages. Therefore, it can better represent the property of IL. However, existing literature mainly concerns predictors of the continuity and satisfaction of online IL from a technical perspective (Kear et al., 2014; Ge et al., 2017), neglecting the relationship between online IL and innovation. To address this gap, this study will explore the influence of teachers' online IL on innovative teaching. Given preceding discussion, we offer:

*Hypothesis 1*: Online informal learning has a direct effect on innovative teaching performance.

### Teaching Efficacy and Autonomous Motivation as the Mediators Between Informal Learning and Innovation

Innovative teaching performance is a presentation of individual innovation in the education field. It refers to the innovative or creative behavior that teachers purposely apply to teaching content, method, and student evaluation, intending to guide students to explore knowledge and develop their creativity (Zhang and Zhang, 2012). Innovative teaching is a new teaching method that differs from the traditional pattern of knowledge-transfer teaching (Wang et al., 2016). Those who cannot promote the innovative development of students are not teaching innovatively, although they are novel and effective, such as teaching by imparting knowledge, constraining students thinking, and even encouraging surface learning. In this study, it includes innovative teaching ideation, action, and outcome, representing teachers' instruction of students' learning strategies, the inspiration of students' motivation, evaluation of students' ideas, encouragement of students' flexibility, etc. (Zhang et al., 2008) Prior studies show that teaching efficacy, innovative efficacy, and intrinsic and autonomous motivation (AM) are important predictors of innovative teaching (Wang et al., 2010; Zhang and Zhang, 2012; Xiong et al., 2020). They are usually based on Bandura's social cognition theory and Deci's self-determination theory (SDT).

Personal teaching efficacy denotes teachers' judgment of their capabilities to achieve desired outcomes of student engagement and learning, including for students with learning difficulties or low motivation (Yu et al., 1995). According to social cognition theory, the environment influences self-competence perceptions and then promotes interest and engagement in activities (Bandura, 1997). In the creative domain, many empirical studies have found that the environment promotes innovation by improving self-efficacy (Zhang and Zhang, 2012; Ma et al., 2013; Jaiswal and Dhar, 2015). For example, domain-relevant and creativity-relevant skills trainings facilitate self-efficacy (Bruce et al., 2010; McDonough et al., 2016; Ding et al., 2020) or innovative efficacy (Byrge and Tang, 2015; Vally et al., 2019) and then improve innovation. For students, informal science learning activities such as visiting science venues improve students' scientific performance through scientific self-efficacy (Suter, 2014; Tang et al., 2019). For teachers, IL positively relates to self-confidence (McCormack et al., 2006; Henze et al., 2009), and teaching efficacy increases innovative teaching (Wang et al., 2010; Xiong et al., 2020). Thus, we offer:

*Hypothesis 2*: Online informal learning has an indirect effect on innovative teaching performance *via* the mediator of personal teaching efficacy.

According to SDT, autonomous motivation (AM) emphasizes individual freedom to engage in certain behaviors in pursuit of personal interests or beliefs; by contrast, controlled motivation is an internal or external pressure (e.g., guilt, demand from others) to engage in a particular behavior (Ryan and Deci, 2000; Gagné and Deci, 2005). Extant literature on AM has suggested an important mediator between environment and performance or innovation. For example, leaders' autonomy support (Taylor et al., 2009; Zhang et al., 2010), teachers' autonomy support (Chen et al., 2015), school atmosphere's autonomy support (Jang et al., 2010; Zhan et al., 2016), and organizational innovation Climate (Zhang and Zhang, 2012) improve the AM of employees, teachers, and students and then increase performance or innovation. Given preceding literature, we offer:

*Hypothesis 3*: Online informal learning has an indirect effect on innovative teaching performance via the mediator of AM.

### A Left Question: the Cognition-Oriented Motivation in Informal Learning and Innovation

Prior studies explore how self-efficacy and motivation separately relate to innovation (Scott and Bruce, 1994; De Jesus et al., 2013; Liu D. et al., 2016), neglecting the relationship between efficacy and motivation. There is not yet a clear and concrete understanding of how they work together in theory and practice. According to the self-efficacy theory, the higher the individual's self-efficacy, the more innovatively he/she will behave. Bandura (1997) speculates that this is because people with high self-efficacy will have high interest and devotion, which drives their persistence to overcome difficulties. Interest, devotion, and persistence in Bandura's speculation all reflect the properties of motivation. According to his view, maybe efficacy influences motivation and then behaviors. However, there is a lack of empirical research, which is helpful to understand how and why self-efficacy affects innovation.

SDT is one of the fundamental motivation theories, which reveals the relationship between efficacy and AM. It puts forward two cognition processes of an environment promoting AM (i.e., cognitive evaluation theory). One is self-determined: external events such as choice opportunity and democratic participation in work can make individuals feel self-determined, which will enhance their AM. The other is competence belief: when the external events make the individual feel competent for the job, leading to the change of competence belief, his/her AM will be enhanced. In addition, compared with the two aspects mentioned earlier, the sense of belonging is suggested by SDT as the remote facilitator. Further empirical SDT studies have indicated that an environment that satisfies an individual's three psychological needs of self-determined, competence, and belonging increases his/her intrinsic motivation and facilitate the internalization of extrinsic motivation (i.e., AM), leading ultimately to enhanced performance, innovation, or well-being (Deci et al., 2001; Richer et al., 2002; Zhang et al., 2010; Devloo et al., 2015; Cai and Gong, 2019). In these studies, three psychological needs satisfaction (autonomy, competence, and relationship) and AM are sequential mediators between environment support and individual behaviors. The competence need satisfaction means changes of competence judgment, which is similar to efficacy belief. Thus, we assume that in the autonomous learning context, an individual's self-efficacy belief may be the antecedent of AM.

In addition, the other research stream puts forward a point of view of social cognition-oriented motivation, which explains the relationship between efficacy and motivation. According to expectancy–value theory (Wigfifield and Eccles, 2000), individual behavior choices and achievements are influenced by a series of factors sequenced as follows: social world, cognitive process, and motivational beliefs. For example, whether individuals have motivations for a task depends on his/her series of cognition, including judgment on task value, his/her competence and need, etc., which are from his/her interactions with the social world (perception of and support from social incidents). Thus, self-belief constructs (e.g., self-efficacy, self-concept, self-esteem, and self-confidence) are considered important antecedents of motivation (Wigfifield and Eccles, 2000; Cetin-dindar, 2015; Doménech-Betoret et al., 2017). Studies have explored the positive impact of learning efficacy on adolescents' learning outcomes (achievement, satisfaction, and online learning performance) through learning motivation or value and expectations (Doménech-Betoret et al., 2017; Duan and Hong, 2019). These studies have enriched the social cognition perspective of motivation, but the results are still limited (Williams, 2010; Doménech-Betoret et al., 2017).

Indeed, there is an alternative explanation that the increase in AM through IL may lead to self-efficacy and thus cause innovation. But it should be supported by other corresponding theories instead of those mentioned earlier, and future research is needed to contribute new theory. Given preceding discussion, we test the following hypothesis:

*Hypothesis 4*: Online informal learning has an indirect effect on innovative teaching performance *via* the sequential mediators of personal teaching efficacy and AM.

This research aims to explore a new phenomenon appearing during the COVID-19 pandemic: online IL and innovation. We speculate that teachers online IL positively influences innovative teaching performance through arousing motivation, which is based on IL's property of self-directed behavior. This perspective helps to deepen the interaction perspective of prior research, explaining why and how interactions through IL increase innovation. This study will verify the cognition-oriented motivation theory. Its novelty focuses on the motivational mechanism, which is important to clarify the process of self-constructing motivation in IL.

## Methods

### Participants and Procedure

We adopted a random questionnaire survey method, taking college teachers from three nationwide teaching communities as the research sample. The teaching communities are online networks organized by the state and universities for training, in which some teachers are pioneers of IL, some are observers, and others are followers. Because IL is a voluntary choice, no one can guarantee that every teacher is an informal learner whose learning frequency, depth, and width are all random. Therefore, this sample is random. A total of 479 faculty participated in the study during July 2020. Participation was voluntary. Before filling the questionnaire, the participants completed a written informed consent form, which is approved by the first author's University Survey Research Ethics Committee. The authors administered the questionnaire and data. The sample comprised 182 males (38%) and 297 females (62%). In terms of teaching experience, 134 (28%) had taught for 10 years or less, 200 (41.8%) for 11–20 years, 145 (30.3%) for 21 years or more. Concerning their discipline background, 114 (23.8%) taught science, 145 (30.3%) taught engineering, and 220 (45.9%) taught humanities and social sciences. The sample included 48 (17.5%) teachers from key research-oriented institutions, 292 (61%) from teaching-oriented provincial institutions, and 103 (21.5%) from vocational institutions. Concerning educational level, 75 (15.7%) teachers had a bachelor's degree, 240 (50.1%) had a master's degree, 161 (33.6%) had a doctoral degree, and three teachers did not report their academic qualifications.

### Measures

The investigation instructions in this study asked faculty to respond to items based on their learning and teaching experience over the previous 5 months of the epidemic (February–July 2020).

### Informal Learning

The frequency of college faculty engagement in online IL activities was measured using the Informal Learning Scale, which is developed and revised by the authors of this study, with reference to other Informal Learning Scales about primary and middle school teachers (Bakkenes et al., 2010; Huang et al., 2020). The scale contains seven items. Responses were given on a five-point Likert scale ranging from “basically no” (1) to “often” (5). Sample items include “observing and exchanging teaching techniques and methods”; “observing and exchanging specific teaching pattern (flipped classroom, blended online, and offline teaching, etc.)”; “learning and exchanging educational idea and theory”; “participating in a seminar on specific teaching problems (e.g., how to instruct students' cooperative learning and evaluate them in the context of online teaching)”; “exchanging and discussing faculty development issues”; “exchanging and discussing student development issues”; “participating in an interdisciplinary seminar (epidemic development, international situation, etc.).” Its Cronbach's alpha coefficient is 0.888.

### Personal Teaching Efficacy

The Personal Teaching Efficacy Scale (PTES) consists of six items, and it was adapted from Teacher Efficacy Scale (Yu et al., 1995). Data for the PTES were self-reported and rated using a five-point Likert scale, which ranged from (1) “totally inconsistent” to (5) “totally consistent.” Examples of PTES were as follows: “I have confidence in my ability to solve teaching problems”; “I have my own effective method in teaching”; “I have ways to promote students' learning effect and interest in learning.” Its Cronbach's alpha coefficient is 0.904.

### Autonomous Motivation

The AM was measured using a subscale of the Motivation at Work Scale, which was revised by Gagné et al. (2010). Participants rated each item on a five-point Likert scale from (1) “totally inconsistent” to (5) “totally consistent.” The subscale consists of six items, and sample items include: “I like teaching very much”; “Teaching work enables me to achieve my life goals”; “I can have a lot of fun when doing teaching work.” Its Cronbach's alpha coefficient is 0.699, approximately reaching the threshold of 0.7 (Nunnally, 1978).

### Innovative Teaching Performance

The Innovative Teaching Performance Scale included three dimensions: innovative teaching ideation, innovative teaching action, and innovative teaching outcome, which was adapted from the Teacher Innovative Work Behavior Questionnaire (Zhang and Zhang, 2012). The 16 items with five items for innovative teaching ideation, six items for innovative teaching action, and five items for innovative teaching outcome were rated on a five-point Likert-type scale from (1) “totally inconsistent” to (5) “totally consistent.” Sample items include “I pay attention to feedback information related to teaching in students' homework” (ideation); “I actively organize teaching activities to enhance students' interest” (action); “I have encouraged students to propose new solutions to problems” (action); and “In my lectures, students have made innovative achievements (reports, products, programs, activities) (outcome).” Its Cronbach's alpha coefficient is 0.921.

### Data Analysis Strategy

All data were analyzed using SPSS 22.0 and Amos 23. First, the descriptive statistics (M and SD) and Pearson's correlations between variables were calculated in SPSS to provide a preliminary test of Hypothesis 1. Meanwhile, a confirmatory factor analysis (CFA) was conducted to examine the construct validity for each scale by Amos. Second, we used the PROCESS macro (Model 6) in SPSS to analyze the serial mediating role to test Hypotheses 2, 3, and 4. In analyzing the results, 95% confidence intervals (CIs) not including zero are taken to indicate a statistically significant mediation effect (Hayes, 2013).

## Results

### Common Method Variance Test

We used Harman's single factor test to check common method variance. The results indicated that the first factor explained only 36.79% (<40%) of the total variance. Therefore, common method bias was unlikely to be a problem in this study.

### Preliminary Analysis

The descriptive statistics and correlation results of the four variables are displayed in Table 1 (at the bottom of this thesis). As predicted, all four variables positively correlated with each other.

**Table 1:** Descriptive statistics, Cronbach's α, and correlation matrix.

	**1**	**2**	**3**	**4**
1.IL	—	—	—	—
2.PTE	0.44^**^	—	—	—
3.AM	0.23^*^	0.53^**^	—	—
4.ITP	0.55^**^	0.70^***^	0.52^**^	—
*M*	2.56	4.22	4.32	4.29
*SD*	0.65	0.59	0.68	0.52
Cronbach's *alpha*	0.888	0.904	0.699	0.921

* *p < 0.05*,

** *p < 0.01*,

*** *p < 0.001. IL, informal learning; PTE, personal teaching efficacy; AM, autonomous motivation; ITP, innovative teaching performance*.

In terms of construct validity, based on the two-step procedure (Anderson and Gerbing, 1988), we first performed a CFA to test the fitness of the measurement model to the research data before examining the structural relationships among four variables. The measurement model comprised four latent constructs and 22 observed indicators (3 factors of Innovative Teaching and 19 items of other scales). In the CFA, latent constructs were allowed to be freely correlated with each other, and observed indicators were specified to load only on their respective latent constructs. The measurement model results showed an excellent data fit [χ^2^ = 437.362; df = 163;χ^2^/df = 2.683; comparative fit index (CFI) = 0.951; goodness of fit index (GFI) = 0.912; Tucker–Lewis index (TLI) = 0.943; SRMR = 0.044; root mean square error of approximation (RMSEA) = 0.049 (90% CI: 0.043, 0.052)].

We further tested the fitness of two alternative models, including a three-factor model (indicators of teaching efficacy and AM were loaded together on one latent construct) and a one-factor model (all 22 indicators were loaded together on one latent construct). The CFA results for the three-factor model were as follows: χ^2^ = 660.215, df = 164; χ^2^/df = 4.026; CFI = 0.911; GFI = 0.865; TLI = 0.897; SRMR = 0.057; RMSEA = 0.080 (90% CI: 0.073, 0.086). The CFA results for the one-factor model were as follows: χ^2^ = 1982.064; df = 169; χ^2^/df = 11.728; CFI = 0.675; GFI = 0.597; TLI = 0.635; SRMR = 0.122; RMSEA = 0.150 (90% CI 0.144, 0.156). The fit index of both alternative models failed to meet the recommended criteria (Wu, 2009). The results of the chi-square statistic also demonstrated that the measurement model fit the data better than did the three-factor model (Δχ^2^ = 222.853, df = 1, *p* < 0.001) or the one-factor model (Δχ^2^ = 1544.702, df = 6, *p* < 0.001).

### Test of Mediation

**Figure 1:**
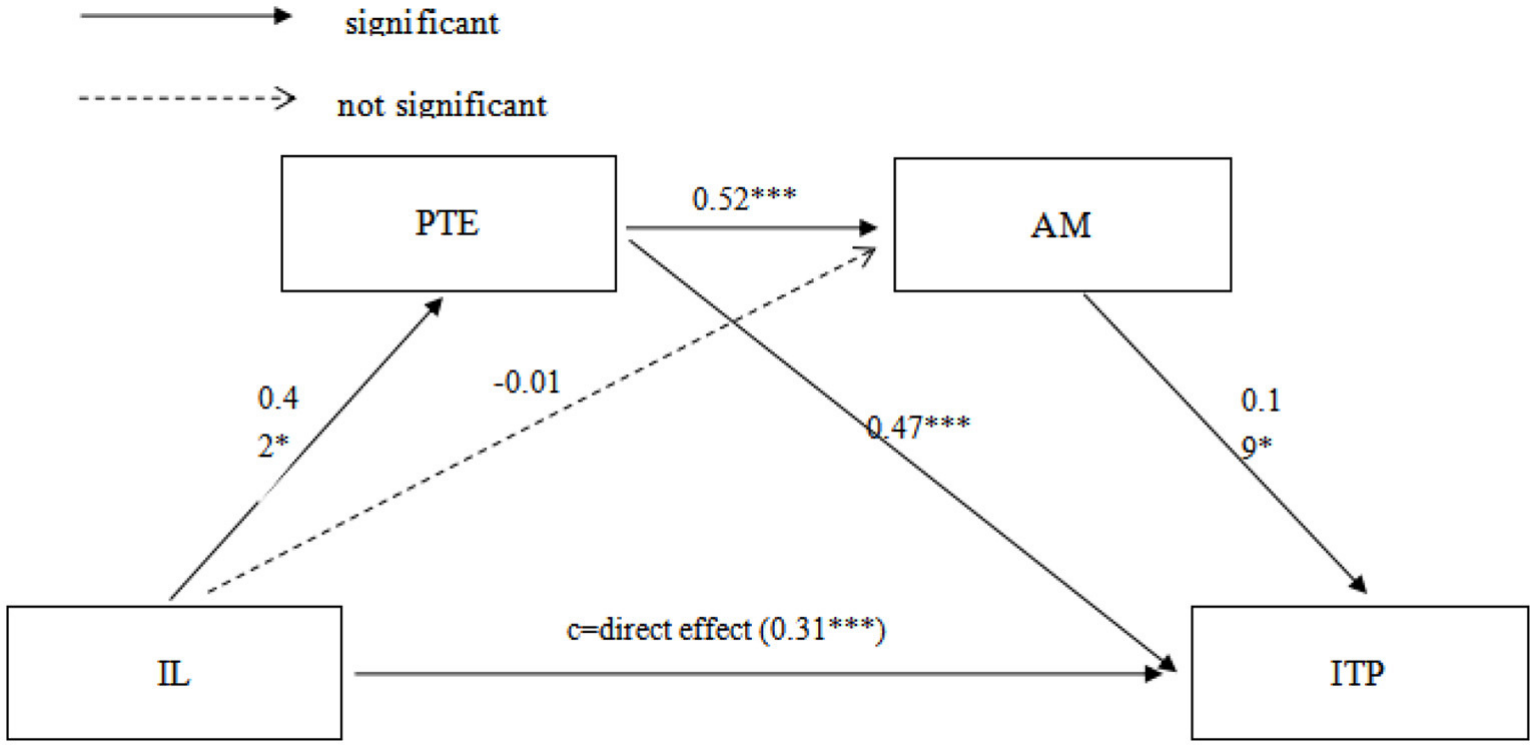
Sequential mediation model regarding the mediating effects of PTE and AM on the relationship between IL and ITP. All the path coefficients were standardized. *N* = 479. IL, informal learning; PTE, personal teaching efficacy; AM, autonomous motivaton; ITP, innovation teaching performance. ***p < 0.0001; *p < 0.05.

We used the Hayes macro PROCESS (Hayes, 2013) to explore the sequential mediation relationship. Personal teaching efficacy and AM were entered as mediators between IL and innovative teaching performance, with gender and years of teaching experience as control variables. The results are presented in Figure 1, Table 2.

**Table 2:** Indirect effects and confidence intervals of mediation analysis, controlling for gender, and teaching age.

**Model pathways**	**Effect value**	**SEB**	**95% CI**	
			**Lower**	**Upper**
Indirect effect1: IL-PTE- ITP	−0.04^α^	0.02	0.15	0.24
Indirect effect2: IL-AM- ITP	−0.01	0.01	−0.02	0.02
Indirect effect3: IL-PTE-AM- ITP	0.04^α^	0.01	0.02	0.07
C1	0.20^α^	0.03	014	0.25
C2	0.15^α^	0.03	0.10	0.21
C3	−0.04	0.01	−0.07	−0.02

*IL, informal learning; PTE, personal teaching efficacy; AM, autonomous motivation; ITP, innovative teaching performance*.

*C1, Indirect effect1 minus Indirect effect2*.

*C2, Indirect effect1 minus Indirect effect3*.

*C3, Indirect effect2 minus Indirect effect3*.

*SEB, bootstrapped standard error; 95% CI, 95% confidence interval*.

α *Empirical 95% confidence interval does not overlap with zero*.

The total effect of IL on innovative teaching performance was significant (β = 0.55, *p* < 0.001). The direct effect of IL on innovative teaching performance was positive and significant (β = 0.31, *p* < 0.001). Thus, hypothesis *H*1 was accepted. The indirect effect of IL on innovative teaching performance through personal teaching efficacy was significant [β = 0.19, bootstrapped standard error (SEB) = 0.02, 95% CI (0.15, 0.24)]. Thus, hypothesis *H2* was supported. However, the indirect effect of IL on innovative teaching performance through AM was not significant [β = −0.01, SEB = 0.01, 95% CI (−0.02, 0.02)]. Hypothesis *H3* was rejected. There was a significant positive indirect effect of IL on innovative teaching performance through personal teaching efficacy and AM [β = 0.04, SEB = 0.01, 95% CI (0.02, 0.07)]. It can be seen that hypothesis *H4* was accepted. These results indicate that personal teaching efficacy and AM partially mediate the relationship between IL and innovative teaching performance. In addition, the results of pairwise contrast of three indirect effects showed significant differences. According to the data results, the mediating effect of indirect effect 1 is greater than indirect effect 2 and indirect effect 3 [β = 0.20, SEB = 0.03, 95% CI (0.14, 0.25); β= 0.15, SEB = 0.03, 95% CI (0.10, 0.21)]. Also, the mediating effect of indirect effect 2 is smaller than that of indirect effect 3 [β = −0.04, SEB = 0.01, 95% CI (−0.07, −0.02)]. Thus, the mediating role of personal teaching efficacy has the biggest effect, and then, the sequential mediation of personal teaching efficacy and AM has the second effect, and mediation of AM has the smallest effect.

## Discussion

The purpose of the current study was to explore the relationship between teachers' online IL and innovative teaching performance through the mediators of personal teaching efficacy and AM.

The direct effect result shows that teachers' online IL has a positive effect on their innovative teaching (supporting *Hypothesis 1*). It reinforces previous findings that innovation can be improved through workplace social interaction (Wenger and Snyder, 2000; Zhou and Lu, 2009; Liu X. Y. et al., 2016) and verifies IL's nature of social constructivism (Watkins and Marsick, 1992; Ellinger, 2004; Marsick et al., 2017). In this research, the content of teachers' online IL is mainly the exchange of teaching experience and problems, and the learning method is to observe, imitate, consult, and reflect. It covers some complex learning patterns, such as problem-based learning, cooperative learning, cognitive apprenticeship learning, etc., recommended by social constructivism learning theory, which insists that learning is not symbolic operations in an individual's mind but an act of coordinating with the environment and other people and then generating knowledge (Vygotsky, 1978; Zhao and Huang, 2011; Cetin-dindar, 2015). In this way, IL constitutes a practice community, where people come together to create a culture based on the discovery of meaning. This constructivist view can integrate prior diffuse findings on interacting predictors of innovation, such as feedback, support, (in)direct experience, workplace networks, etc. (Wenger and Snyder, 2000; Gino et al., 2010; De Stobbeleir et al., 2011; Harrison and Rouse, 2015; Liu X. Y. et al., 2016). As a pioneer exploration, this study contributes by demonstrating the link between IL and innovation and explaining a low-cost and highly convenient new method for inspiring innovation.

**Figure 2:**
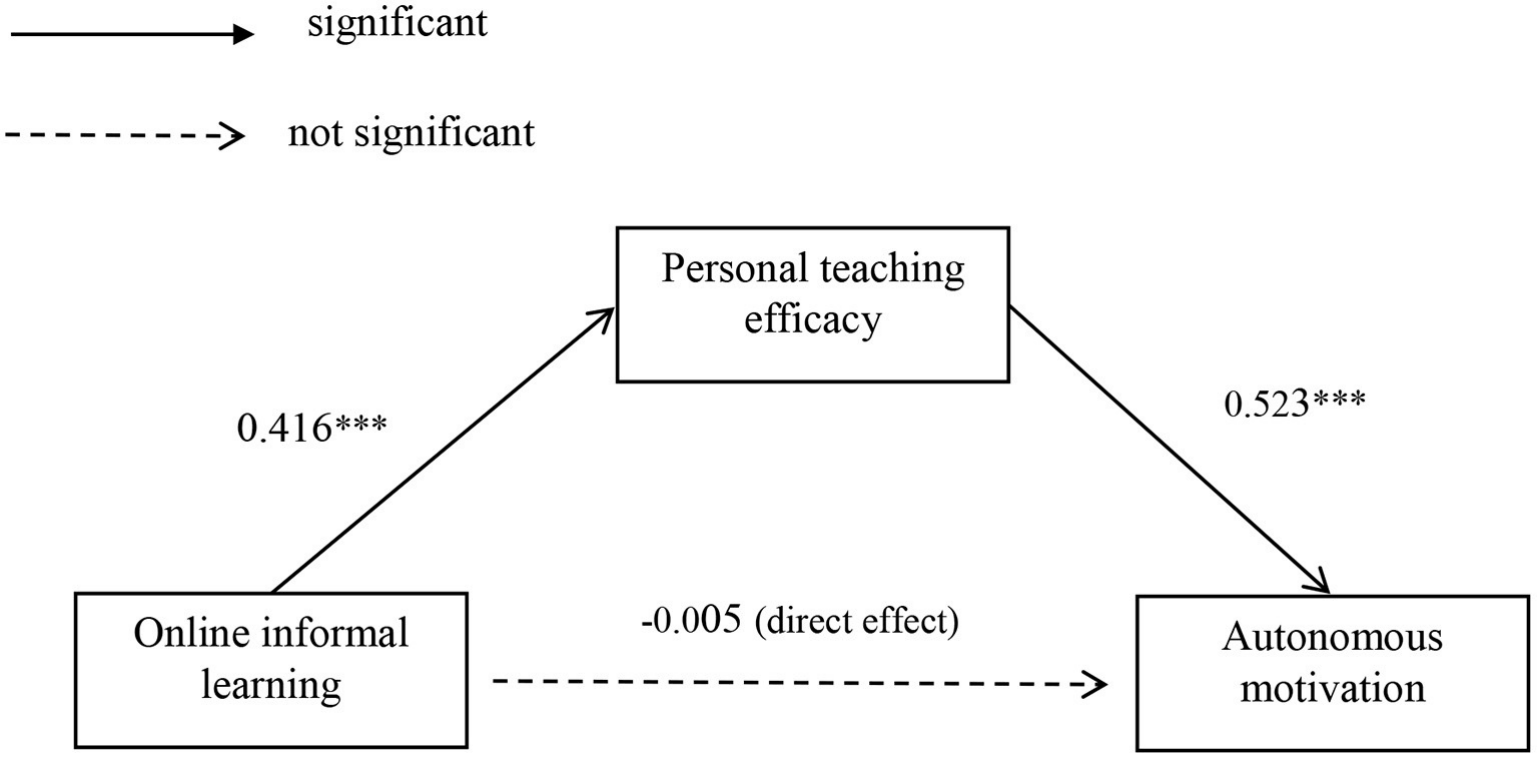
Mediating effects of personal teaching efficacy between online informal learning and autonomous motivation. ***p < 0.001.

The analysis of indirect effects shows that personal teaching efficacy plays an intermediary role between online IL and innovative teaching (supporting *Hypothesis 2*), consistent with prior findings that efficacy mediates between environment (e.g., training, leadership, support, creative atmosphere) and innovation (Tierney and Farmer, 2002; Scott et al., 2004; Gong et al., 2009; Liu D. et al., 2016). Notably, the indirect effect of IL on innovative teaching performance through AM was not significant (rejecting *Hypothesis 3*). Similarly, the direct effect of IL on AM was not significant either in the integrative model, although the correlation between these two variables was significant (*r* = 0.23). To verify the outcome, the relationship among IL, teaching efficacy, and autonomic motivation was further tested in one model. The direct effect of IL on AM was not significant (β = −0.005, *p* > 0.05). The indirect effect of IL on AM was positive and significant (β = 0.416, *p* < 0.001; β = 0.523, *p* < 0.001) (see Table 2, Figure 2). These results suggest that personal teaching efficacy plays a fully mediating role between IL and AM. In other words, extensive IL can foster greater personal teaching efficacy leading to higher AM. According to Amabile, intrinsic motivation (the main part of AM) is more variable and subject to the influence of one's work environment; additionally, it does not appear automatically but needs to be awakened and nurtured (Amabile, 1993). This research verifies this view and shows that only the IL engagement is not enough to increase AM and cause innovation. The changes of efficacy belief through learning is an indispensable antecedent of AM (Hew and Cheung, 2014; Doménech-Betoret et al., 2017; Duan and Hong, 2019).

The sequential mediation analysis result shows that personal teaching efficacy and AM play intermediary roles between online IL and innovative teaching performance (supporting *Hypothesis 4*). This result is consistent with Gagné and Deci's SDT regarding competence and AM as sequential mediators between supportive environment and innovation (Deci et al., 2001; Zhang et al., 2010), but in detail, this result expands on it by identifying self-efficacy as a possible mediator. It is also consistent with cognition-oriented motivation theory, which reinforces that self-efficacy begets motivation in a learning context and plays a motivational role in behaviors (Doménech-Betoret et al., 2017; Duan and Hong, 2019). The findings suggest that extensive IL leads to higher personal teaching efficacy, thereby promoting AM and finally increasing creativity in teaching. The core of IL is the active and autonomous construction in the practice of social interaction. Teachers' process of autonomous construction entails: (1) interacting with other teachers and reflecting on personal discovery and inquiry; (2) acquiring and updating professional knowledge/skills, meanwhile generating education ideals (“what and how I can do”: efficacy); (3) actively seeking a breakthrough in work and enjoying fun in teaching (“what I want to do”: AM); (4) teaching with enthusiasm and by one's own will (practice personal teaching style), and showing a creative vitality and growth (innovation). Furthermore, from the constructivist learning perspective, the process mentioned earlier reveals the formulation of teaching self-identity, i.e., the beliefs of “what I can do,” “what I want to do,” and “how I will do,” and then developing their own teaching styles, which denotes innovative teaching. In sum, IL is not a simple interactive process, just concerning ideas, and methods exchanges. This study highlights its aspect of internal autonomy construction. Without the autonomous construction of teaching self-identity and individualized teaching style, teachers cannot innovate by themselves and keep on innovating. Maybe they sometimes copy few innovative teaching cases from others but cannot last long. This study reveals the internal transmission mechanism of online IL to innovative teaching, highlighting the important role of self-efficacy in constructing an effective online IL pattern.

This study makes two main contributions to the field of IL and innovative teaching within the online context. First, we found that IL is an effective way to promote innovation for its social constructivist learning features and thus extend the IL–innovation relationship literature, which has been under-explored in previous research. Second, we enrich the understanding of the motivation mechanism of innovation from the perspective of cognitive motivation theory. This research proposes a more comprehensive theoretical model: self-efficacy and AM sequentially mediate the relationship between IL and innovation. We also found that the sense of efficacy has a grounded status in this model. The transformation of IL into innovation is based on not only the learning of knowledge and skills but also the real changes of competence belief, which will cause a series of motivation construction processes, e.g., teaching self-identity and individualized teaching style. In sum, our findings reveal the motivation mechanism through which innovation can be driven, and we highlight that one's internal autonomy construction is the key to innovation.

This study also has some limitations that suggest directions for future research. First, we used a cross-sectional design, which does not allow causal inferences. Future experimental or longitudinal designs are needed to confirm our results. Second, we used self-reports to measure innovative teaching performance, which may have introduced response bias. Future studies should use other methods to collect data on innovation, including ratings by leaders and colleagues or creative, experimental tasks. Lastly, participants were all teachers from innovative teaching communities in China, limiting the results' generalizability across other teachers and cultures. We call for future cross-cultural research and studies of other types of professionals.

The findings of this study have some theoretical and practical implications for psychological empowerment. In the era of the rapidly developing Internet, how to activate individuals and help employees empower themselves (e.g., self-energized utility) has become the top priority of organizational management. Psychological empowerment is considered an effective management approach (Thomas and Velthouse, 1990; Chen et al., 2019). According to our results, IL affects innovative teaching through personal teaching efficacy and AM. The mechanism of psychological empowerment in IL is of great research value and needs to be furthered. These findings indicate that innovation might be developed through online IL interventions targeted toward domain-related self-efficacy. Self-efficacy may awaken or cultivate AM and then help individuals to innovate. Thus, psychological empowerment has two meanings: improving individual self-efficacy and stimulating individuals' AM for work.

## Data Availability Statement

The raw data supporting the conclusions of this article will be made available by the authors, without undue reservation.

## Ethics Statement

The studies involving human participants were reviewed and approved by Ethics Committee of School of Education at Huazhong University of Science & Technology. The patients/participants provided their written informed consent to participate in this study.

## Author Contributions

HY designed the research and wrote the manuscript. JZ carried out the design of research ideas. RZ is responsible for statistics and language. All authors contributed to the article and approved the submitted version.

## Conflict of Interest

The authors declare that the research was conducted in the absence of any commercial or financial relationships that could be construed as a potential conflict of interest.

## References

[B1] Abecassis-Moedas C. Sguera F. Ettlie J. E. (2016). Observe, innovate, succeed: a learning perspective on innovation and the performance of entrepreneurial chefs. J. Bus. Res. 69, 2840–2848. 10.1016/j.jbusres.2015.12.053

[B2] Amabile T. M. (1993). Motivational synergy: toward new conceptualizations of intrinsic and extrinsic motivation in the workplace. Hum. Resour. Manage. Rev. 3, 185–201. 10.1016/1053-4822(93)90012-S

[B3] Amabile T. M. (1996). Creativity in Context. New York, NY: Westview Press.

[B4] Anderson J. C. Gerbing D. W. (1988). Structural equation modeling in practice: a review and recommended two-step approach. Psychol. Bull. 103, 411–423. 10.1037/0033-2909.103.3.411

[B5] Bakkenes I. Vermunt J. D. Wubbels T. (2010). Teacher learning in the context of educational innovation: learning activities and learning outcomes of experienced teachers. Learn. Instruct. 20, 533–548. 10.1016/j.learninstruc.2009.09.001

[B6] Bandura A. (1997). Self-Efficacy: The Exercise of Control. New York, NY: Freeman.

[B7] Bruce C. D. Esmonde I. Ross J. Dookie L. Beatty R. (2010). The effects of sustained classroom-embedded teacher professional learning on teacher efficacy and related student achievement. Teach Teacher Educ. 26, 1598–1608. 10.1016/j.tate.2010.06.011

[B8] Byrge C. Tang C. (2015). Embodied creativity training: effects on creative self-efficacy and creative production. Think. Skills Creativ. 16, 51–61. 10.1016/j.tsc.2015.01.002

[B9] Cai Y. H. Gong J. (2019). The relationship between school support and teachers' teaching innovation: the mediating role of basic psychological needs satisfaction. J. Educ. Stud. 48–57. 10.14082/j.cnki.167398.2019.02.007

[B10] Cetin-dindar A. (2015). Student motivation in constructivist learning environment. Eurasia J. Mathemat. Sci. Technol. Educ. 12, 233–247. 10.12973/eurasia.2016.1399a

[B11] Chen J. W. Guo Y. Y. Hu X. Y. (2015). Effect of autonomous motivation and family's social class on the relationship between teacher's autonomy support and junior middle school students' academic engagement. Psychol. Dev. Educ. 2, 180–187.

[B12] Chen L. S. Shen W. Z. Zhen W. B. Xu S. H. (2019). Review on psychological empowerment in 30 years in the context of self-energized utility. Hum. Resour. Dev. China 36, 37–52.

[B13] China's Ministry of Education. (2020). Information About Online Education in Colleges and Universities and Consideration of the Next Work. Retrieved from: http://www.moe.gov.cn/fbh/live/2020/51987/sfcl/202005/t20200514_454117.html (accessed May 14, 2020).

[B14] Cunningham J. Hillier E. (2013). Informal learning in the workplace: key activities and processes. Educ. Train. 55, 37–51. 10.1108/00400911311294960

[B15] De Jesus S. N. Rus C. L. Lens W. Imaginário S. (2013). Intrinsic motivation and creativity related to product: a meta-analysis of the studies published between 1990–2010. Creativ. Res. J. 25, 80–84. 10.1080/10400419.2013.752235

[B16] De Stobbeleir K. E. M. Ashford S. J. Buyens D. (2011). Self-regulation of creativity at work: the role of feedback-seeking behavior in creative performance. Acad. Manage. J. 54, 811–831. 10.5465/amj.2011.64870144

[B17] Deci E. L. Ryan R. M. Gagné M. Leone D. R. Usunov J. Kornazheva B. P. (2001). Need satisfaction, motivation, and well-being in the work organizations of a former Eastern Bloc country. Personal. Soc. Psychol. Bullet. 27, 930 – 942. 10.1177/0146167201278002

[B18] Devloo T. Anseel F. De Beuckelaer A. Salanova M. (2015). Keep the fire burning: reciprocal gains of basic need satisfaction, intrinsic motivation and innovative work behaviour. Eur. J. Work Organiz. Psychol. 24, 491–504. 10.1080/1359432X.2014.931326

[B19] Ding D. Brinkman W.-P. Neerincx M. A. (2020). Simulated thoughts in virtual reality for negotiation training enhance self-efficacy and knowledge. Int. J. Hum. Comput. Studies 139:102400. 10.1016/j.ijhcs.2020.102400

[B20] Doménech-Betoret F. Abellán-Roselló L. Gómez-Artiga A. (2017). Self-efficacy, satisfaction, and academic achievement: the mediator role of students'expectancy-value beliefs. Front. Psychol. 8:1193. 10.3389/fpsyg.2017.01193 28769839 PMC5513915

[B21] Duan Z. H. Hong J. Z. (2019). The relationship between internet teacher-student interaction and online learning performance: the mediating effect of internet learning self-efficacy and internet learning motivation. Psychol. Dev. Educ. 35, 58–65.

[B22] Ellinger A. D. (2004). The concept of self-directed learning and its lmplications for human resource development. Adv. Dev. Hum. Resour. 6, 158–177. 10.1177/1523422304263327

[B23] Eraut M. (2004). Informal learning in the workplace. Studies Continuing Educ. 26, 247–273. 10.1080/158037042000225245

[B24] Flynn D. Eddy E. R. Tannenbaum S. I. (2006). The impact of national culture on the continuous learning environment: exploratory findings from multiple countries. J. East-West Business 12, 85–107. 10.1300/J097v12n02_05

[B25] Gagné M. Deci E. L. (2005). Self-determination theory and work motivation. J. Organiz. Behav. 26, 331–362. 10.1002/job.322

[B26] Gagné M. Forest J. Gilbert M.-H. Aube C. Morin E. Malorni A. (2010). The motivation at work scale: validation evidence in two languages. Educ. Psychol. Measure. 70, 628–646. 10.1177/0013164409355698

[B27] Ge N. Meng Z. K. Xu M. D. Zhang Y. C. (2017). Research on the influence factors of social presence in informal Network learning community. Distance Educ. China 504, 39–46.

[B28] George J. M. (2007). Creativity in organizations. Acad. Manage. Ann. 1, 439–477. 10.5465/078559814

[B29] Gino F. Argote L. Miron-Spektor E. Todorova G. (2010). First, get your feet wet: the effects of learning from direct and indirect experience on team creativity. Organiz. Behav. Hum. Decis. Process. 111, 102–115. 10.1016/j.obhdp.2009.11.002

[B30] Gong Y. Huang J.-C. Farh J.-L. (2009). Employee learning orientation, transformational leadership, and employee creativity: the mediating role of employee creative self-Efficacy. Acad. Manage. J. 52, 765–778. 10.5465/amj.2009.43670890

[B31] Harrison S. H. Rouse E. D. (2015). An inductive study of feedback interactions over the course of creative projects. Acad. Manage. J. 58, 375–404. 10.5465/amj.2012.0737

[B32] Hayes A. F. (2013). Introduction to Mediation, Moderation, and Conditional Process Analysis: A Regression-Based Approach (1 edition). New York, NY: The Guilford Press.

[B33] Henze I. van Driel J. H. Verloop N. (2009). Experienced science teachers' learning in the context of educational innovation. J. Teach. Educ. 60, 184–199. 10.1177/0022487108329275

[B34] Hew K. F. Cheung W. S. (2014). Students' and instructors' use of massive open online courses (moocs): motivations and challenges. Educ. Res. Rev. 12, 45–58. 10.1016/j.edurev.2014.05.001

[B35] Huang X. Lee C. K. Frenzel A. C. (2020). Striving to become a better teacher: linking teacher emotions with informal teacher learning across the teaching career. Front. Psychol. 11:1067. 10.3389/fpsyg.2020.01067 32536892 PMC7269101

[B36] Jacobs R. L. Park Y. (2009). A proposed conceptual framework of workplace learning: implications for theory development and research in human resource development. Hum. Resour. Dev. Rev. 8, 133–150. 10.1177/1534484309334269

[B37] Jaiswal N. K. Dhar R. L. (2015). Transformational leadership, innovation climate, creative self-efficacy and employee creativity: a multilevel study. Int. J. Hosp. Manag. 51, 30–41. 10.1016/j.ijhm.2015.07.002

[B38] Jang H. Reeve J. Deci E. L. (2010). Engaging students in learning activities: it is not autonomy support or structure but autonomy support and structure. J. Educ. Psychol. 102, 588–600. 10.1037/a0019682

[B39] Kear K. Chetwynd F. Jefferis H. (2014). Social presence in online learning communities: the role of personal profiles. Res. Learn. Technol. 22, 1–15. 10.3402/rlt.v22.19710

[B40] Koopmans H. Doornbos A. J. Eekelen I. M. V. (2006). Learning in interactive work situations: it takes two to tango; why not invite both partners to dance? Hum. Resour. Dev. Quart. 17, 135–158. 10.1002/hrdq.11669220713

[B41] Kyndt E. Gijbels D. Grosemans I. Donche V. (2016). Teachers' everyday professional development: mapping informal learning activities, antecedents, and learning outcomes. Rev. Educ. Res. 86, 1111–1150. 10.3102/0034654315627864

[B42] Li Y. Y. Zhang H. M. Zhang H. Z. (2020). Model construction and empirical test of college students'satisfaction with online learning during epidemic prevention and control period: based on the survey of 15 universities in Shanghai. Open Educ. Res. 26, 112–111.

[B43] Liu D. Jiang K. Shalley C. E. Keem S. Zhou J. (2016). Motivational mechanisms of employee creativity: a meta-analytic examination and theoretical extension of the creativity literature. Organiz. Behav. Hum. Decision Proces. 137, 236–263. 10.1016/j.obhdp.2016.08.001

[B44] Liu X. Y. Li X. J. Sun J. Q. (2016). Research on relationship between community of practice participation and employee innovative behavior—moderating effect of feedback-seeking and perceived organizational support. Sci. Technol. Manage. Res. 20, 130–136+189.

[B45] Louws M. L. Meirink J. A. Van Veen K. Van Driel J. H. (2017). Teachers'self-directed learning and teaching experience: what, how, and why teachers want to learn. Teach. Teach. Educ. 66, 171–183. 10.1016/j.tate.2017.04.004

[B46] Ma Y. Cheng W. Ribbens B. A. Zhou J. (2013). Linking ethical leadership to employee creativity: knowledge sharing and self-efficacy as mediators. Soc. Behav. Pers. 41, 1409–1419. 10.2224/sbp.2013.41.9.1409

[B47] Manuti A. Pastore S. Scardigno A. F. Giancaspro M. L. Morciano D. (2015). Formal and informal learning in the workplace: a research review. Int. J. Train. Dev. 19, 1–17. 10.1111/ijtd.12044

[B48] Marsick V. J. Watkins K. E. Scully-Russ E. Nicolaides A. (2017). Rethinking informal and incidental learning in terms of complexity and the social context. J. Adult Learn. Knowl. Innovat. 1, 27–34. 10.1556/2059.01.2016.003

[B49] McCormack A. Gore J. Thomas K. (2006). Early career teacher professional learning. Asia-Pacific J. Teach. Educ. 34, 95–113. 10.1080/13598660500480282

[B50] McDonough K. Crimlisk J. Nicholas P. Cabral H. Quinn E. K. Jalisi S. (2016). Standardizing nurse training strategies to improve knowledge and self-efficacy with tracheostomy and laryngectomy care. Appl. Nursing Res. 32, 212–216. 10.1016/j.apnr.2016.08.003 27969030

[B51] Nunnally J. C. (1978). Psychometric Theory (2nd ed.). New York, NY: McGraw-Hill.

[B52] OECD (2020). A Framework to Guide An Education Response to the COVID-19 Pandemic of 2020[EB/OL]. Available online at: https://read.oecd-ilibrary.org/view/?ref=126_126988-t63lxosohs&title=A-frame-work-to-guide-an-education-response-to-the-Covid-19-Pandemic-of-2020

[B53] Quezada R. L. Talbot C. Quezada-Parker K. B. (2020). From bricks and mortar to remote teaching: a teacher education programme's response to COVID-19. J. Educ. Teach. 46, 472–483. 10.1080/02607476.2020.1801330

[B54] Richer S. F. Blanchard C. Vallerand R. J. (2002). A motivational model of work turnover. J. Appl. Soc. Psychol. 32, 2089–2113. 10.1111/j.1559-1816.2002.tb02065.x

[B55] Rowden R. W. Conine C. T. (2005). The impact of workplace learning on job satisfaction in smallus commercial banks. J. Workplace Learn. 17, 215–230. 10.1108/13665620510597176

[B56] Ryan R. M. Deci E. L. (2000). Self-determination theory and the facilitation of intrinsic motivation, social development, and well-being. Am. Psychol. 55, 68–78. 10.1037/0003-066X.55.1.68 11392867

[B57] Scott G. Leritz L. E. Mumford M. D. (2004). The effectiveness of creativity training: a quantitative review. Creativ. Res. J. 16, 361–388. 10.1080/10400410409534549

[B58] Scott S. G. Bruce R. A. (1994). Determinants of innovative behavior: a path model of individual innovation in the workplace. Acad. Manage. J. 37:580. 10.2307/256701

[B59] Suter L. E. (2014). Visiting science museums during middle and high school: a longitudinal analysis of student performance in science. Sci. Educ. 98, 815–839. 10.1002/sce.21116

[B60] Tang X. Zhang D. H. Zhong J. C. (2019). Influence of informal science learning on science achievement: mediating effect of scientific interest and self-efficacy. J. Shanghai Educ. Res. 5, 58–62.

[B61] Taylor I. M. Ntoumanis N. Smith B. (2009). The social context as a determinant of teacher motivational strategies in physical education. Psychol. Sport Exercise 10, 235–243. 10.1016/j.psychsport.2008.09.002 18369244

[B62] Thomas J. C. (2004). A review of informal learning literature, theory and implications for practice in developing global professional competence. J. Europ. Industrial Train. 28, 283–295. 10.1108/03090590410527663

[B63] Thomas K. W. Velthouse B. A. (1990). Cognitive elements of empowerment: an “Interpretive” model of intrinsic task motivation. Acad. Manage. Rev. 15, 666–681. 10.5465/amr.1990.4310926

[B64] Tian R. Xiong Z. Y. Normand Romuald. (2020). Challenges and solutions in teaching and learning in the COVID-19 crisis:analysis and reflection based on OECD's “a framework to guide an education response to COVID-19 pandemic of 2020”. J. Distance Educ. 38, 3–14. 10.15881/j.cnki.cn33-1304/g4.2020.04.001

[B65] Tierney P. Farmer S. M. (2002). Creative self-efficacy: its potential antecedents and relationship to creative performance. Acad. Manage. J. 45, 1137–1148. 10.5465/3069429

[B66] Vally Z. Salloum L. AlQedra D. El Shazly S. Albloshi M. Alsheraifi S.. (2019). Examining the effects of creativity training on creative production, creative self-efficacy, and neuro-executive functioning. Think Skills Creativ. 31, 70–78. 10.1016/j.tsc.2018.11.003

[B67] Vygotsky L. S. (1978). Mind in Society: The Development of Higher Mental Process. Cambridge, MA: Harvard University Press.

[B68] Wang H. C. Liu J. Y. Xie D. B. (2016). Innovative teaching in universities: ideas, characteristics and mistakes. China Univ. Teach. 19–23.

[B69] Wang Z. H. Wang K. J. You X. Q. Dang H. X. (2010). Effects of efficacy, work motivation, and mood on teaching innovation. Psychol. Sci. 33, 1254–1257. 10.16719/j.cnki.1671-6981.2010.05.057

[B70] Watkins K. E. Marsick V. J. (1992). Towards a theory of informal and incidental learning in organizations*. Int. J. Lifelong Educ. 11, 287–300. 10.1080/0260137920110403

[B71] Wenger E. C. Snyder W. M. (2000). Communities of practice: the organizational frontier. Harvard Business Rev. 78, 139–145.

[B72] Wigfifield A. Eccles J. S. (2000). Expectancy-value theory of achievement motivation. Contemp. Educ. Psychol. 25, 68–81. 10.1006/ceps.1999.1015 10620382

[B73] Williams D. M. (2010). Outcome expectancy and self-efficacy: theoretical implications of an unresolved contradiction. Pers. Soc. Psychol. Rev. 14, 417–425. 10.1177/1088868310368802 20505161

[B74] Wolfson M. A. Tannenbaum S. I. Mathieu J. E. Maynard M. T. (2018). A cross-level investigation of informal field-based learning and performance improvements. J. Appl. Psychol. 103, 14–36. 10.1037/apl0000267 28933909

[B75] Wu M. L. (2009). Structural Equation Model: Operation and Application of AMOS. Chongqing: Chongqing University Press.

[B76] Xiong Y. Sun X. Y. Liu X. Q. Wang P. Zheng B. (2020). The influence of self-efficacy and work input on physical education teachers'creative teaching. Front. Psychol. 10:2856. 10.3389/fpsyg.2019.02856 31993003 PMC6964797

[B77] Yu G. L. Xin T. Shen J. L. (1995). Teacher's sense of teaching efficacy: it's structure and influencing factors. Acta Psychol. Sinica 27, 159–166.

[B78] Yu S. Q. Mao F. (2005). Informal learning: a new field of e-learning research and practice. e-Educ. Res. 10, 18–23.

[B79] Zhan Y. S. Li M. L. Zhang Y. (2016). An empirical study on the relationship between engineer in undergraduate learning motivation and autonomy-support education environment. Res. Higher Educ. Eng. 6, 25–31.

[B80] Zhang J. Zhang W. Feng J. (2010). Relationships between Leaders' autonomy support and creative performance of employee. China Soft Sci. 62–69.

[B81] Zhang J. H. Chu Y. X. Lin C. D. (2008). Construct of test on creative teaching behavior in teachers. Psychol. Dev. Educ. 3, 109–114.

[B82] Zhang M. Zhang L. (2012). Teacher's innovative work behavior and innovation climate. Chinese J. Ergonom. 18, 1–6.

[B83] Zhao C. Gao Z. H. (2017). The dynamic evolution and mechanism of newcomer's political self-efficacy: an interactionist perspective of organizational socialization. Adv. Psychol. Sci. 25, 1456–1468. 10.3724/SP.J.1042.2017.01456

[B84] Zhao M. R. Huang X. H. (2011). Analysis of teacher learning from the perspective of constructivism. Educ. Res. 373, 83.

[B85] Zhou G. H. Lu L. J. (2009). A positive study on impact extent of informal network learning upon innovation performance of cluster enterprise. Sci. Sci. Manage. S.and T. 30, 74–77.

